# Conservative Treatment of Complicated Crown Fracture and Crown-Root Fracture of Young Permanent Incisor—A Case Report with 24-Month Follow-Up

**DOI:** 10.3390/children8090725

**Published:** 2021-08-25

**Authors:** David Marinčák, Vojtěch Doležel, Michal Přibyl, Iva Voborná, Ivo Marek, Jiří Šedý, Radovan Žižka

**Affiliations:** 1 Institute of Dentistry and Oral Sciences, Medical Faculty, University of Palacký, 772 00 Olomouc, Czech Republic; vojtech.dolezel@upol.cz (V.D.); michal.pribyl@upol.cz (M.P.); iva.voborna@upol.cz (I.V.); ortho.marek@gmail.com (I.M.); jirisedy@jirisedy.cz (J.Š.); radovan.zizka@upol.cz (R.Ž.); 2 Department of Anatomy, Second Faculty of Medicine, Charles University, 150 06 Prague, Czech Republic

**Keywords:** pulpotomy, tooth fracture, tooth injury

## Abstract

The complicated crown-root fracture of young permanent teeth is an uncommon traumatic dental injury that is usually treated in a complex way and is demanding not only for the dentist but even for the treated child. In this case report, we present the conservative treatment of a maxillary central incisor in a 10-year-old boy after a traumatic dental injury. Treatment included partial pulpotomy and adhesive fragment reattachment after reflection of the mucoperiosteal flap. The patient was fully asymptomatic at 24-month follow-up, with an aesthetically acceptable outcome. Vital pulp therapy and adhesive fragment reattachment can be a viable treatment option for complicated crown-root fractures, especially when treating immature permanent teeth.

## 1. Introduction

Dental trauma is a frequent occurrence in permanent dentition and can occur at any age, with increased numbers documented in the first and second decades of life [1]. When considering the spectrum of injuries, crown fractures with or without pulp exposure are the most common type, varying from 26.2% to 44.1% of all dental injuries. Crown-root fractures with or without pulp exposure are the opposite, being the least frequent types of dental trauma, accounting for 0.56% to 1.1% of all dental traumatic injuries [1,2,3]. For crown fractures, there are relatively easy and well-documented treatment possibilities that vary based on the involvement of pulp. In uncomplicated crown fractures, if the fragment is saved, the simple adhesive reattachment of the fragment is the method of choice. If the fragment is lost or unsuitable for reattachment, then direct resin composite restoration is preferred [4]. If the pulp is exposed, vital pulp therapy such as pulp capping or partial or deep pulpotomy is preferred to conventional root canal treatment [5]. For crown-root fractures, the treatment is even more demanding, considering that the fragment margin is subgingival in the majority of cases. Several treatment possibilities including resin composite restoration with a supragingival margin of restoration, crown lengthening, orthodontic or surgical extrusion, or fragment reattachment have been described [6]. Numerous clinical studies suggest that adhesive reattachment of the coronal fragment can be a valuable treatment modality for crown-root-fractured teeth [7,8]. This approach can potentially preserve the pulp and reduce treatment time and costs, since it preserves the natural tooth and leads to a natural aesthetic outcome. Thus far, long-term studies investigating the clinical outcomes of fragment reattachment in complicated crown-root fractures are lacking. There is growing evidence that adhesive fragment reattachment is a suitable long-term solution after complicated or uncomplicated crown fracture [9,10,11], or crown-root fracture in general [12].

Despite this growing evidence, there are only case reports or case series for the treatment of complicated crown-root fractures, which were recently summarized in a systematic review [13]. Importantly, in all (except one) of these published cases, root canal treatment was performed [14], indicating that root canal treatment is the standard procedure in this type of tooth fracture. Moreover, in 85% of cases, a post was used as part of the restoration. However, in immature permanent teeth with pulp exposure of traumatic origin, vital pulp treatment is recommended and should be preferred to root canal treatment [15,16]. In these particular scenarios, vital pulp treatment has a high success rate, reduced time requirements [17], and in comparison to root canal treatment, it has better cost-effectiveness [18].

The present case report aims to show a conservative treatment possibility after complicated crown-root fracture in an immature permanent central incisor that consists of partial pulpotomy and adhesive reattachment of the fragment.

## 2. Case Presentation

In February 2019, a 10-year-old Caucasian boy presented one day after trauma during ice skating and sustained a complicated crown fracture of tooth 11 and complicated crown-root fracture of tooth 21. The fragment of tooth 11 was found immediately after the traumatic dental injury and was stored in mineral water. The patient and his guardian promptly visited his general practitioner, who performed splinting from self-cure resin from tooth 53 to 63, performed direct pulp capping, and referred the patient to our department for further treatment.

### 2.1. Examination of Patient

**Figure 1:**
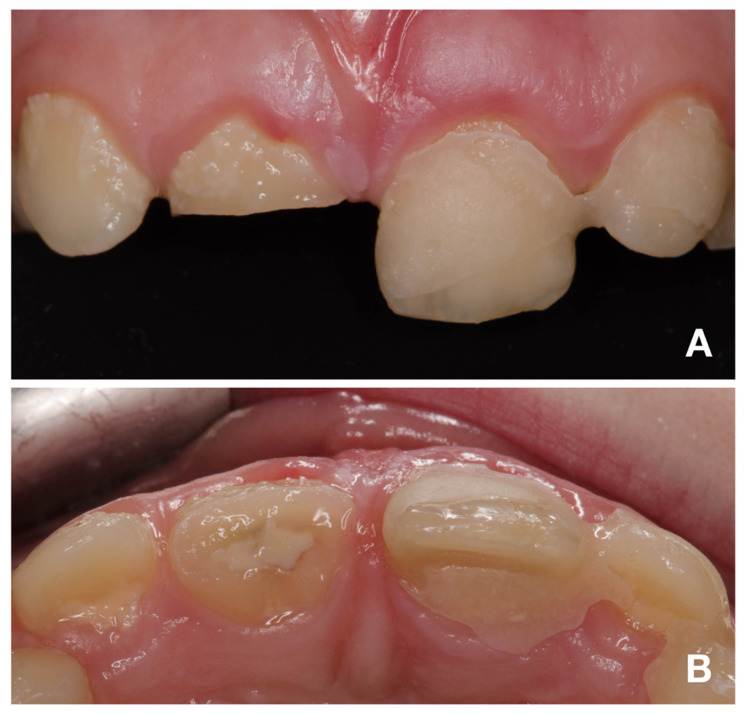
Intraoral photographs of teeth 12–22. (**A**)—buccal view; (**B**)—occlusal view.

**Figure 2:**
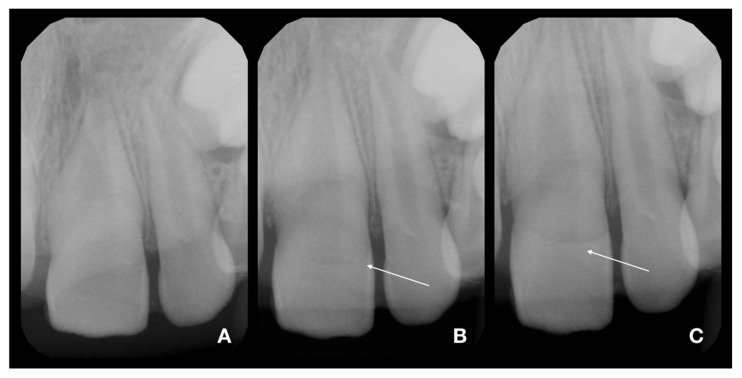
Diagnostic X-rays of teeth 21 and 22 in three different angulations [21]. (**A**)—foreshortened X-ray (increased angulation of central beam to the direction used in ordinary occlusal exposure). The fracture line is almost not visible. (**B**)—distortion-free X-ray (angulation used in ordinary occlusal exposures). The fracture line started to be pronounced; see arrow. (**C**)—elongated X-ray (decreased angulation of central beam to the direction used in ordinary occlusal exposure). The fracture line is clearly visible; see arrow.

**Figure 3:**
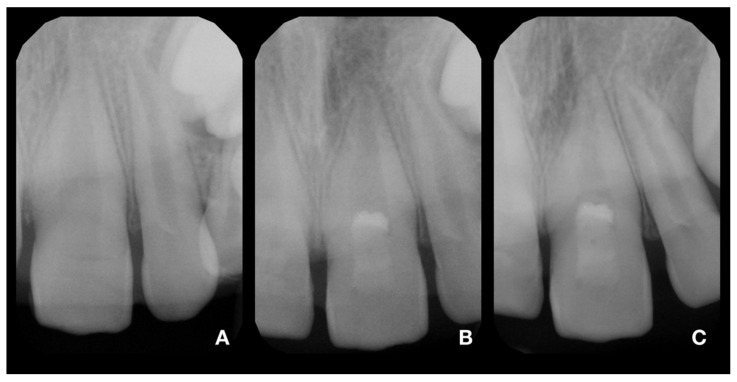
X-rays of tooth 21. (**A**)—diagnostic X-ray of tooth 21; (**B**)—postoperative X-ray of tooth 21; (**C**)—control X-ray at 24-month follow-up. The continuing development of the root and narrowing of the root canal is visible. In addition, the formation of a radiopaque bridge under the calcium silicate cement and the absence of periapical radiolucency are apparent.

**Figure 4:**
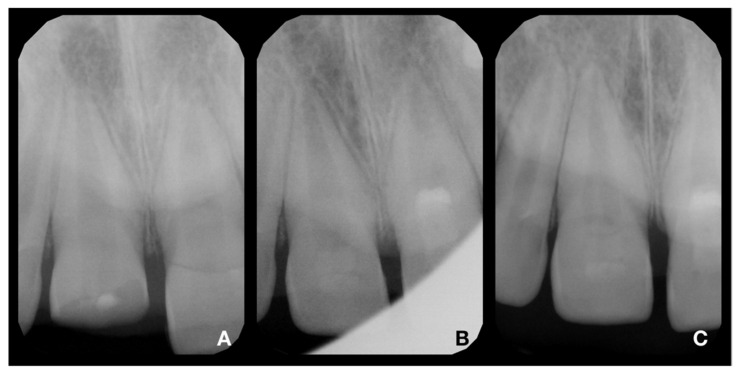
X-rays of tooth 11. (**A**)—diagnostic X-ray of tooth 11; (**B**)—postoperative X-ray of tooth 11; (**C**)—control X-ray at 24-month follow-up. The continuing development of the root and narrowing of the root canal is visible. In addition, the formation of a radiopaque bridge under the calcium silicate cement and the absence of periapical radiolucency are apparent.

An intraoral examination showed the presence of splinting only from tooth 21 to 63 and a complicated fracture of tooth 11 (Figure 1A). The patient mentioned the partial loss of splinting within a short time after emergency treatment. In the maxillary right central incisor, the fracture was oblique, extending in the apical direction from the labial to palatal surface. The margin on the palatal surface was 2 mm above the marginal gingiva (Figure 1B). The maxillary left central incisor displayed first-grade mobility even with the presence of splinting, but the patient denied any discomfort or pain. The percussion of the upper incisor was not painful, as well as the palpation of alveolar bone. The thermal sensitivity (cold) tests were positive for both central incisors and comparable to adjacent teeth, so a pulpal diagnosis of normal pulp was made. The diagnostic X-rays showed the presence of crown-root fracture (class III according to Ellis classification of dental traumatic injuries [19]) of immature tooth 21 (class IV according to Cvek’s classification of root development [20]), which was located subgingivally all around; therefore, it was not possible to locate the exact path of the fracture line (Figure 2 and Figure 3A). The diagnostic X-ray of immature tooth 11 (class IV according to Cvek’s classification of root development [20]) showed no tooth dislocation (Figure 4A). The guardian was informed about the clinical situation and about the preliminary treatment plan, which consisted of partial pulpotomy and fragment reattachment of tooth 11 and extraction of the coronal fragment of tooth 21 to assess the possibilities of further treatment, since it was impossible to locate the fracture line due to the subgingival location. Informed consent was obtained.

### 2.2. Partial Pulpotomy and Adhesive Reattachment of Tooth 11

**Figure 5:**
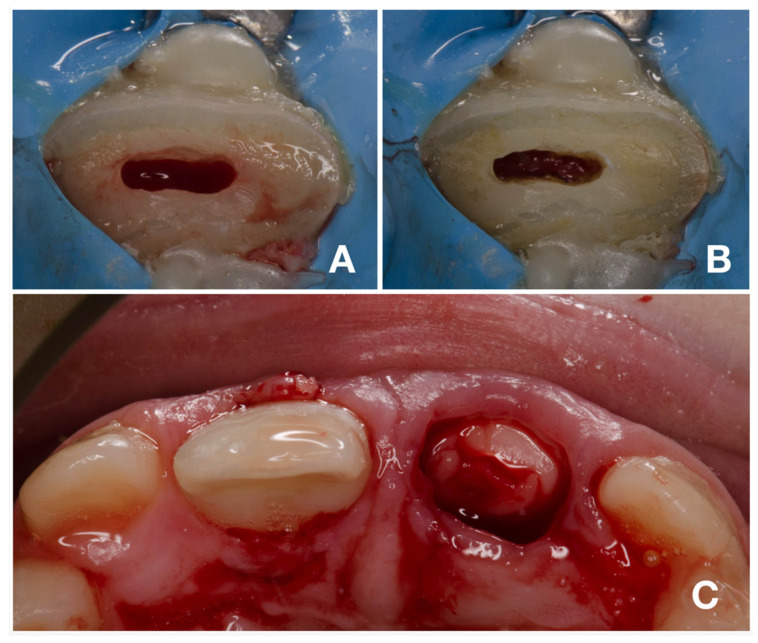
Partial pulpotomy and adhesive fragment reattachment of tooth 11. (**A**)—clinical situation immediately after pulpotomy; (**B**)—clinical situation after achieving hemostasis; (**C**)—occlusal view after adhesive reattachment and removal of coronal fragment of tooth 21.

While the patient was under local anesthesia with 1.7 mL of Ubistesin (3M ESPE GmbH, Seefeld, Germany), a rubber dam was applied solely on tooth 11. After partial pulpotomy of 1 mm of dental pulp (Figure 5A), hemostasis was achieved within 2 min with the use of 1% sodium hypochlorite (Figure 5B). Due to concerns about the possible future discoloration of a clinical crown, calcium silicate cement with a low tendency to discoloration was used (Biodentin, Septodont, Saint-Maur-des-Fossés, France). After compaction of Biodentin with paper points, the tooth was cleaned with a microbrush with saline solution, and the exposed dentin and enamel were carefully sandblasted with 50 µm aluminum oxide. The total-etch technique was used for the fragment and tooth. For this purpose, 36% phosphoric acid gel (DeTrey Conditioner 36, Dentsply Sirona GmbH, Bensheim, Germany) and a multi-bottle adhesive system (Optibond FL, Kerr GmbH, Biberach, Germany) were used. The flow resin composite (Filtek Ultimate flow, 3M ESPE GmbH, Seefeld, Germany) was used as an intermediate material. Subsequently, the excess material was removed, and the transition was polished.

### 2.3. Deep Pulpotomy and Adhesive Reattachment of Tooth 21 after Flap Reflection

**Figure 6:**
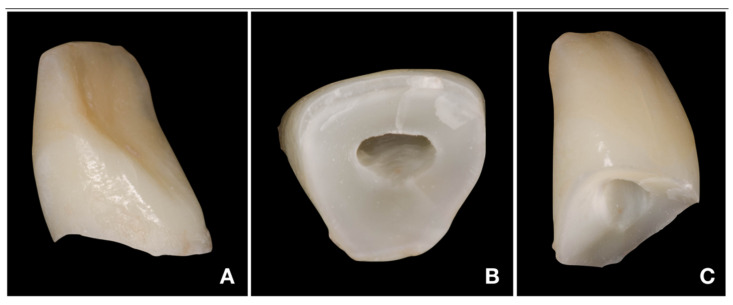
Fragment of tooth 21. (**A**)—distal side of fragment with clearly visible location of fracture line apically to cementoenamel junction; (**B**)—cervical side of fragment with enlarged pulp chamber; (**C**)—mesial side of fragment.

**Figure 7:**
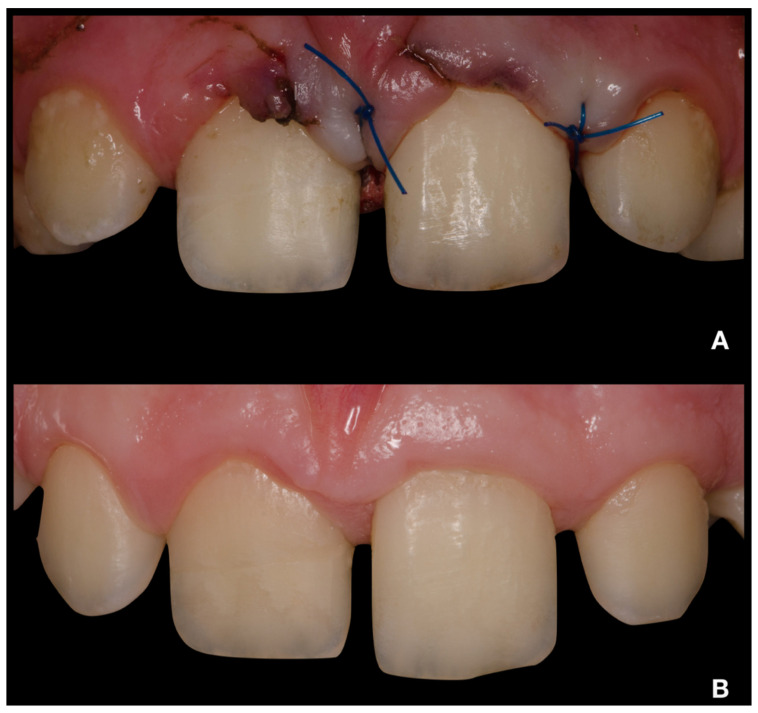
Intraoral photographs of teeth 12–22 from buccal view. (**A**)—immediately after treatment; (**B**)—24-month follow-up.

**Figure 8:**
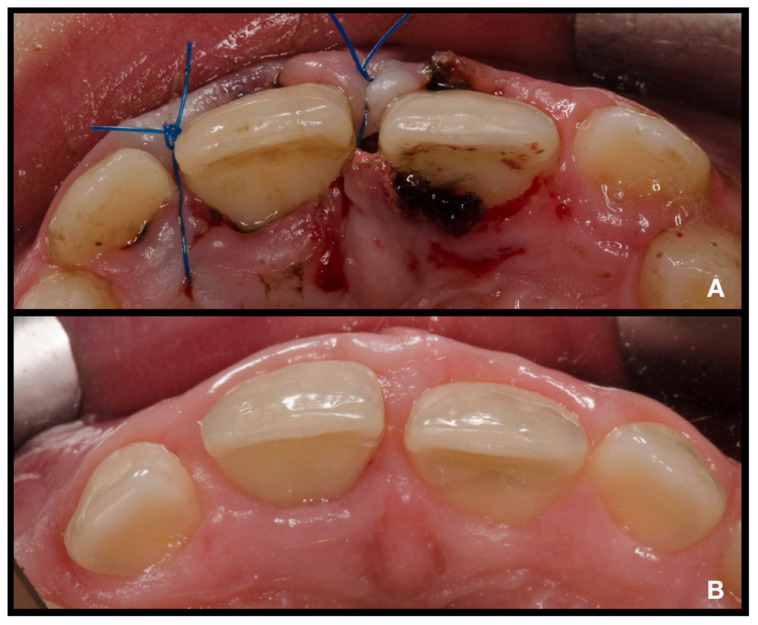
Intraoral photographs of teeth 12–22 from occlusal view. (**A**)—immediately after treatment; (**B**)—24-month follow-up.

After adhesive reattachment of tooth 11, the coronal fragment of tooth 21 was extracted (Figure 5C), and the course of the fracture line was evaluated. On the extracted fragment, the location of the fracture line was clearly visible down to the cementoenamel junction on most of the circumference (Figure 6A). The enamel was present only on the buccal aspect of the crown (Figure 6B). The palatal margin of the tooth was probed and was found to be located approximately 1 mm above the crestal bone. After consultation with the patient’s guardian, we agreed on vital pulp therapy and adhesive fragment reattachment of tooth 21 after reflection of the mucoperiosteal flap. Before the surgical procedure, the pulp chamber of the fragment was cleaned and extended to enhance the surface for intermediate material and bonding (Figure 6B,C). The full-thickness mucoperiosteal flap was raised after intrasulcular incisions from tooth 11 to tooth 22. Since it was impossible to apply a rubber dam, Viscostat clear (Ultradent, South Jordan, UT, USA) was used to achieve complete hemostasis, enabling proper adhesive conditions for adhesive reattachment. The surroundings of tooth 21 were caulked with Teflon tape to seal the margin of the fracture line. This improved the visibility in the operation field and enabled a further shift of the raised mucoperiosteal flap. The partial pulpotomy of 2 mm of dental pulp was performed, and hemostasis was achieved almost immediately with the use of 1% sodium hypochlorite. Due to the location of a coronal barrier deep under the gingival margin, we were not concerned about the possible discoloration, and we thus chose a material with better handling properties—MTA plus (Cerkamed, Stalowa Wola, Poland). After compaction of MTA plus with paper points, the tooth was cleaned with a microbrush and the surface was carefully sandblasted with 50 µm aluminum oxide. For adhesive reattachment of tooth 21, the same adhesive protocol as for the adhesive reattachment of tooth 11 was used. The total-etch technique was used for the fragment and as well as the tooth. For this purpose, 36% phosphoric acid gel (DeTrey Conditioner 36, Dentsply Sirona GmbH, Bensheim, Germany) and a multi-bottle adhesive system (Optibond FL, Kerr GmbH, Biberach, Germany) were used. The flow resin composite (Filtek Ultimate flow, 3M ESPE GmbH, Seefeld, Germany) was used as an intermediate material. The use of Teflon tape also prevented the contamination of the crestal bone with the adhesive system and excess flow composite. The reattachment was cured for 120 s from different sides to allow the proper setting of the composite. After the removal of excess material and polishing of the transition, suturing two simple knots with monofilament was performed (Figure 7A). Immediately after treatment, the laceration of a gingiva and loss of soft tissue between central incisors were visible (Figure 7A and Figure 8A). The control X-ray showed proper alignment of fragments and the rest of both central incisors (Figure 3B and Figure 4B). Both patient and parent were advised regarding post-operative care in order to achieve optimal healing: to prevent further injury, the patient was advised to avoid participation in contact sports, maintain a soft diet for one week, ensure meticulous oral hygiene, and rinse with an antibacterial agent such as chlorhexidine gluconate 0.12%.

### 2.4. Follow-Up

Clinical and radiological follow-up was performed after 1, 3, 6, 12, 18, and 24 months (Figure 3C, Figure 4C, Figure 7B and Figure 8B). During the follow-up period, the patient was fully asymptomatic; teeth 11 and 21 responded to the thermal sensitivity test positively, despite their threshold being higher than for adjacent teeth. On X-rays, the formation of a non-homogenous dentinal bridge under calcium silicate cement was visible. The root continued its development, including the thickening of the root canal walls. There were no signs of apical radiolucency. The clinical crowns of central incisors did not present any sign of discoloration caused by the calcium silicate cement, and the patient with his parents were pleased with the aesthetic outcome. The probing depth was within physiological limits, with no clinical attachment loss. The free palatal gingiva of tooth 21 was slightly livid, with bleeding on probing. The patient and his parents were informed about the ideal follow-up regime, consisting of clinical and radiological evaluation yearly at least for another 3 years.

## 3. Discussion

Although vital pulp therapy is often used in fragment reattachment after complicated crown fractures [10,22], it is only rarely used in fragment reattachment after complicated crown-root fractures [13]. This case report describes the vital pulp treatment of an immature permanent tooth with subsequent adhesive reattachment of the crown-root fractured fragment after mucoperiosteal flap reflection. Most adhesive reattachment cases after crown-root fracture consist of root canal treatment and post placement [13]. Only one of the published case reports, which also describes vital pulp therapy in the reattachment of a crown-root fractured fragment, is comparable to the clinical situation that we report here [14]. In contrast to our case, this was a mature tooth with point size pulp exposure and the margin was slightly subgingival, where it was possible to place a rubber dam after raising the flap.

Adhesive fragment reattachment is based on the bonding of the fractured fragment back to its original position [22]. Although various reattachment techniques have been described to achieve a larger adhesive surface, simple fragment reattachment with an intermediate material is the preferred procedure [11]. The success rate of adhesive reattachment in crown fractures has risen since the development of modern adhesive systems, from 55% [23] to 84–93% in 2-years follow-up [9,10]. The influence of vital pulp therapy on the success rate is questionable, and the results of clinical studies are contradictory [9,10]. The success rate of adhesive reattachment in crown-root fractures was reported to be as high as 56.4% in a 9.5-year horizon [12]. The main technical difficulty is achieving an operation field suitable for adhesive reattachment with no contamination, which could have deteriorated the adhesive layer. In this study, it was found that adhesive fragment reattachment in deep crown-root fractured teeth was generally associated with mild gingival inflammation. Corresponding to the subgingival fracture location, the periodontal probing depth and clinical attachment loss were significantly higher compared to control teeth, as was the frequency of bleeding on probing. In our case, there was no increase in the probing depth, and there was no clinical attachment loss. This might have been due to the slight supraosseal location of the fracture and further eruption of tooth 21. The clinical attachment loss was reduced because of the reflection of a mucoperiosteal flap instead of gingivectomy, which was often used by Soliman et al. [12].

Unfortunately, there are no scientific data on the occurrence of pulp necrosis after vital pulp therapy and adhesive fragment reattachment. The overall incidence of pulp necrosis after complicated crown fracture is influenced by the occurrence of concomitant luxation injury, the presence of immature root development, and the absence of a response to sensitivity tests immediately after trauma [9,24,25]. Generally, it is suggested to proceed with vital pulp therapy in immature permanent teeth after dental traumatic injury with exposed pulp [15,16], except for intrusion or avulsion [26]. Clinical and histological findings confirm that partial pulpotomy can be used as a permanent treatment modality for mature and immature permanent teeth with complicated crown fractures [27]. The reported success rate of partial pulpotomy in permanent teeth with complicated crown fractures ranges from 87.5% to 100% [27,28]. We can expect a similar success rate for complicated crown-root fractures if the placement of the pulp capping material and its sealing are successful.

One of the reasons behind the deteriorated aesthetic outcome could be discoloration of the tooth [23], which can be caused by the use of calcium silicate cements such as MTA. Their potential for discoloration is influenced by the content of bismuth oxide, as materials with bismuth oxide have higher discoloration potential [29]. In the case of tooth 11, where the calcium silicate cement was aimed to be placed in the coronal part, there were concerns about possible discoloration. Therefore, calcium silicate with a lower potential for discoloration was used [30]. On the other hand, in the case of tooth 21, even though only partial pulpotomy was performed, the coronal barrier was noticeably subgingival, so the concerns about possible discoloration were not significant. Furthermore, in this clinical scenario, with a reflected mucoperiosteal flap, we needed a material with better handling properties in order to reduce the treatment time. Biodentin (Septodont, Saint-Maur-des-Fossés, France) has very low wash-out resistance in comparison to other calcium silicate cements [31], and even minor contamination with any liquid leads to wash-out of the material. For these reasons, the MTA-based calcium silicate cement was used. An appropriate choice of pulp capping material reduces the treatment time and the possibility of discoloration.

During the follow-up regime, it is possible encounter complications, such as pulp necrosis or fragment detachment. After pulp necrosis, the choice of treatment is quite straightforward, and root canal treatment is the first treatment option. In the case of fragment detachment, the decision making is much more complicated. The first possibility is to proceed with repeated adhesive fragment reattachment, which is a viable treatment option [9]. If it is not possible to perform repeated reattachment, root canal treatment and prosthodontic restoration would be the gold-standard treatment. In this situation, the ferrule would be inadequate, and its renewal would demand orthodontic or surgical extrusion, which would result in a compromised crown–root ratio. Another treatment possibility is root submersion, extraction, or autotransplantation [32]—these are much more costly and time-demanding in comparison with partial pulpotomy and adhesive fragment reattachment.

## 4. Conclusions

Vital pulp therapy and adhesive fragment reattachment can be a viable treatment option for complicated crown-root fractures, especially when treating immature permanent teeth. The reflection of the mucoperiosteal flap could lead to lower clinical attachment loss when compared to gingivectomy. Calcium silicate cement with a higher potential to discoloration can be used in deep pulpotomy with an acceptable aesthetic result.

## Data Availability

Not applicable.
